# A Novel Microbiological Method in Microtiter Plates for Screening Seven Kinds of Widely Used Antibiotics Residues in Milk, Chicken Egg and Honey

**DOI:** 10.3389/fmicb.2019.00436

**Published:** 2019-03-12

**Authors:** Qin Wu, Dapeng Peng, Qianying Liu, Muhammad Abu Bakr Shabbir, Abdul Sajid, Zhenli Liu, Yulian Wang, Zonghui Yuan

**Affiliations:** ^1^National Reference Laboratory of Veterinary Drug Residues (HZAU) and MOA Key Laboratory for the Detection of Veterinary Drug Residues, Huazhong Agricultural University, Wuhan, China; ^2^MOA Laboratory for Risk Assessment of Quality and Safety of Livestock and Poultry Products, Huazhong Agricultural University, Wuhan, China; ^3^College of Veterinary Sciences and Animal Husbandry, Abdul Wali Khan University Mardan, Mardan, Pakistan

**Keywords:** antibiotics residues, microbiological inhibition method, *Geobacillus stearothermophilus var* C953, milk, chicken egg, honey

## Abstract

A broad-spectrum microbiological inhibition method has been developed for rapidly screening different kinds of antibiotics such as β-lactam, aminoglycosides, tetracyclines, sulfonamides, macrolides, lincosamides and quinolones in milk, chicken egg and honey by using an easy sample preparation. The microbiological system in microtiter plates consists of an agar medium, a mixture of nutrients, test bacteria (*Geobacillus stearothermophilus var* C953), bromocresol purple, and other supplements such as trimethoprim, chloramphenicol, streptomycin and enrofloxacin which helps to improve the detection capability of the microbiological system toward the chosen antibiotics. It was observed that the limit of detection of the kit used in present study for all kinds of antibiotics in milk were lower than or close to maximum residue limits determined by EU or CODEX. For chicken egg and honey, the detection capability of the kit was similar to that determined in milk. Moreover, it was revealed that the kit in present study was more sensitive to aminoglycosides, macrolides and quinolones in various matrixes than internationally available commercial kits. The false-positive and false-negative rates for both were 0%. The coefficient of variations among various factors was all less than 4%. Additionally, the quality guarantee period of the kit was more than 6 months at 4°C. A good correlation between the kit results and the LC–MS/MS results for milk was also observed, which revealed that the kit was reliable to screen antibiotics residues in incurred samples.

## Introduction

The petri dish and test tube methods are the two sub types of microbiological inhibition methods. Compared to petri dish methods, the test tube methods are more suitable for high-throughput screening of antimicrobial drugs residues in animal food because it is neither time consuming nor laborious (Nagel et al., 2013). *Geobacillus stearothermophilus* is the most widely used indicator bacterium in microbiological inhibition methods in terms of test tubes, as it is not easily contaminated, demands high incubation temperature (55°C) and grows faster in a short time (less than 4 h) than other bacteria. Moreover, it is more sensitive to antimicrobial agents, particularly, β-lactam (Kumar et al., 2012). Additionally, spores of *G. stearothermophilus* are more resistant to adverse factors than vegetative cells and show stable activity for a long time. Therefore, spores of *G. stearothermophilus* can be added into kit’s medium during the kits preparation process, which simplifies the detection procedure and prolongs the shelf life of kits. However, *G. stearothermophilus* is not sensitive enough to many commonly used antibiotics except β-lactam (Nagel et al., 2012). In past years, a number of studies by microbiological inhibition methods in terms of test tubes were developed to improve the sensitivity of *G. stearothermophilus* to different kinds of antibiotics residues in milk. There are brilliant black reduction test (BRT AIM) (Molina et al., 2003), Copan milk test (Le Breton et al., 2007), Doveltest SP-NT (Althaus et al., 2003), Eclipse100^®^ (Beltrán et al., 2015), and Charm^®^ Blue-Yellow II (Linage et al., 2007). Among these kits, Charm^®^ Blue-Yellow II can detect more antibacterial drugs including β-lactam, aminoglycosides, tetracyclines, sulfonamides, and macrolides. However, this method is not sensitive enough to aminoglycosides and macrolides, and extremely insensitive to quinolones. The chicken egg and honey are also consumed daily and important for human health. However, little research by microbiological inhibition methods in terms of test tubes is known about chicken egg and honey. Even Premi^®^Test, the test tube method is widely applied for the detection of antibiotics residues in milk, muscle, kidney, egg, honey and feed etc. However, Premi^®^ Test is not considered ideal to detect residual antibiotics in chicken egg and honey, as it does not show enough sensitivity to aminoglycosides, macrolides and quinolones (Stead et al., 2004). Therefore the aim of the present study was to develop a new test tube method with *G. stearothermophilus var* C953, which was more sensitive to a different kind of antimicrobial agents especially aminoglycosides, macrolides and quinolones in milk, chicken egg, and honey.

## Materials and Methods

### Antimicrobial Standards

β–lactam: penicillin G (PEN), cefquinome (CEF); aminoglycosides: neomycin (NEO), streptomycin (STR); tetracyclines: doxycycline (DOX), tetracycline (TET); macrolides: erythromycin (ERY), spiramycin (SPI); sulfonamides: sulfadiazine (SDZ), sulfadimidine (SDM); lincosamides: lincomycin (LIN); quinolones: danofloxain (DAN), enrofloxacin (ENR); trimethoprim (TMP); and chloramphenicol (CAP) were all purchased from Sigma-Aldrich (St. Louis, MO, United States). Drugs for the preparation of antimicrobial solutions were stored and handled according to the manufacturers’ instructions before use. In addition, the methods for the preparation of stock solutions and working standard solutions of antibiotics were shown in Table 1.

**Table 1:** Methods for the preparation of stock solutions and working standard solutions of antibiotics.

Antimicrobial agents	Solvents	Diluents
β –lactams	Phosphate buffer, pH 6.0, 0.1 mol/L	Phosphate buffer, pH 6.0, 0.1 mol/L
Aminoglycosides	Tris, pH 8.0, 0.01 mol/L	Tris, pH 8.0, 0.01 mol/L
Tetracyclines	HCl, 0.1 mol/L	Phosphate buffer, pH 6.0, 0.1 mol/L
Macrolides	Phosphate buffer, pH 8.0, 0.01 mol/L	Phosphate buffer, pH 8.0, 0.01 mol/L
Sulfonamides	NaOH, 0.1 mol/L	Sterilized distilled water
Lincosamides	Phosphate buffer, pH 8.0, 0.01 mol/L	Phosphate buffer, pH 8.0, 0.01 mol/L
Quinolones	NaOH, 0.1 mol/L	Phosphate buffer, pH 8.0, 0.1 mol/L
TMP	Glacial acetic acid	Sterilized distilled water
CAP	Methanol	Sterilized distilled water


### Test Organism

*Geobacillus stearothermophilus var* C953 was obtained from American Type Culture Centre (ATCC), Rockville, MD, United States.

### Recovery, Preparation and Conservation of Test Organism

A freeze-dried strain of *G. stearothermophilus var* C953 was dissolved in sterile physiological saline (0.85% NaCl). A 100 μL of *G. stearothermophilus var* C953 suspension was inoculated into nutrient agar with 0.035 g/L MnSO_4_ ⋅ H_2_O and incubated in incubator for 24 h at 55°C. After three generations recovery, a single culture from nutrient agar with 0.035 g/L MnSO_4_⋅ H_2_O was inoculated into a new same medium and incubated in incubator for 72 h at 55°C. At the end of incubation, the cells were washed from medium by 10% (v/v) dried skimmed milk. After collection, the cells suspension was dispended into amber vials. Aliquots of cell suspensions stored at 4, -20, and -80°C for 6 h respectively step by step. After that, the frozen cells suspension was freeze-dried by freeze vacuum dryer and stored at -80°C until usage.

### Preparation of Kit’s Medium Components

Plate Count Agar (Becton Dickinson) fortified with glucose (6 g/L; Sigma^®^) was used. The medium was sterilized at 121°C for 15 min. After the medium was cool down to 50 ± 1°C, its pH was adjusted to 7.8 ± 0.1. After that, *G. stearothermophilus var* C953 spore suspension (5 × 10^9^ CFU/L), along with bromocresol purple (0.1 mg/L, Mallinckrodt^®^) and sensitizers such as 50 μg/L trimethoprim (TMP), 40 μg/L chloramphenicol (CAP), 45 μg/L streptomycin (STR) and 60 μg/L enrofloxacin (ENR) were added. A 150 μL of medium was added into each well of microtiter plates by using an electronic pipette (Eppendorf Research^®^Pro) after kit’s medium components mixed well. Finally, these microtiter plates were sealed with aluminized film and conserved at 4°C until use.

### Control Samples

Milk samples were collected from the dairy farm of Huazhong Agricultural University (HZAU), Wuhan, Hubei, China. At the time of samples collection, the cows did not receive any antimicrobial substances in the last 9 weeks and were at postpartum stage (between 60 and 90 days). Because bovine milk presented normal values of chemical composition, total bacterial counts (CFU < 100,000 mL^-1^) and somatic cell counts (SCC < 400,000 mL^-1^) (Debayle et al., 2008) during these days. Milk samples were kept at 4°C for approximately 2 days throughout the experiment. The chicken eggs were collected from laying hens (30 weeks old) with a history of no antimicrobial drugs used either in the form of treatment or growth promoter in last 6 weeks at the chicken farm of HZAU. And chicken eggs were kept at 4°C within 1 week before use. Honey samples were purchased from the local bee farmer and the absence of any antimicrobial substances was confirmed by high performance liquid phase tandem mass spectrometry (Debayle et al., 2008). Moreover, honey samples were stored at 4°C for less than 1 week before use.

### Spiked Samples

Spiked samples were prepared from the respective antibiotics working standard solutions in a single step using antimicrobial drugs-free respective antibiotics diluents, milk, homogeneous eggs and diluted honey (spiked levels see Tables 2–5). In addition, eight concentrations at different levels were prepared for each drug, and 24 replicates were prepared for each concentration.

**Table 2:** Limit of detection (LODs) of microbiological system in antimicrobial agents’ diluents (3.75 h).

Antimicrobial agents	Spiked levels /(μg/L)	EU/CODEX MRL in milk^1,2^/(μg/L)	This kit /(μg/L)
Penicillin G	0, 1, 2, 3, 4, 5, 6, 8	4	2
Cefquinome	0, 2.5, 5, 10, 20, 40, 60, 80	20	20
Neomycin	0, 25, 50, 75, 100, 150, 200, 300	1500	50
Streptomycin	0, 50, 100, 200, 250, 500, 750, 1000	200	200
Doxycycline	0, 25, 50, 75, 100, 150, 200, 300	0	50
Tetracycline	0, 50, 75, 100, 200, 250, 300, 400	100	100
Erythromycin	0, 10, 20, 30, 40, 50, 75, 100	40	40
Spiramycin	0, 50, 75, 100, 200, 250, 300, 400	200	200
Sulfadiazine	0, 25, 50, 75, 100, 150, 200, 300	100	50
Sulfadimidine	0, 50, 75, 100, 200, 250, 300, 400	100	100
Lincomycin	0, 25, 50, 75, 100, 150, 200, 300	150	150
Danofloxain	0, 50, 75, 100, 200, 250, 300, 400	30	100
Enrofloxacin	0, 50, 100, 180, 200, 220, 250, 280	100	180


*^1^ (The European Commission (2010)). ^*2*^Food, 2015*.

**Table 3:** LODs of microbiological system in milk (3 h).

Antimicrobial agents	Spiked levels /(μg/L)	EU/CODEX MRL in milk^1,2^ /(μg/L)	This kit /(μg/L)
Penicillin G	0, 1, 2, 3, 4, 5, 6, 8	4	2
Cefquinome	0, 10, 20, 30, 35, 40, 45, 50	20	40
Neomycin	0, 25, 50, 75, 100, 150, 200, 300	1500	50
Streptomycin	0, 50, 100, 200, 220, 250, 280, 300	200	200
Doxycycline	0, 25, 50, 75, 100, 150, 200, 300	0	100
Tetracycline	0, 100, 200, 250, 300, 320, 350	100	300
Erythromycin	0, 10, 20, 30, 40, 50, 75, 100	40	40
Spiramycin	0, 50, 100, 200, 220, 250, 280, 300	200	200
Sulfadiazine	0, 25, 50, 75, 100, 150, 200, 300	100	150
Sulfadimidine	0, 100, 200, 250, 300, 320, 350	100	300
Lincomycin	0, 25, 50, 75, 100, 120, 150, 180	150	120
Danofloxain	0, 50, 75, 100, 200, 250, 300, 400	30	100
Enrofloxacin	0, 100, 200, 300, 400, 430, 450, 480	100	400


*^1^ (The European Commission (2010)). ^*2*^Food, 2015*.

**Table 4:** LODs of microbiological system in chicken egg (3.5 h).

Antimicrobial agents	Spiked levels /(μg/L)	EU/CODEX MRL in chicken egg^1,2^ /(μg/L)	This kit /(μg/L)	Premi^®^ Test (Stead et al., 2004) /(μg/L)
Penicillin G	0, 1, 2, 3, 4, 5, 6, 8	-	4	<2.5
Cefquinome	0, 2.5, 5, 10, 20, 40, 60, 80	-	40	/
Neomycin	0, 25, 50, 75, 100, 150, 200, 300	500	100	/
Streptomycin	0, 50, 100, 200, 250, 500, 750, 1000	-	200	/
Doxycycline	0, 25, 50, 75, 100, 150, 200, 300	-	100	200
Tetracycline	0, 50, 75, 100, 200, 250, 300, 400	200	300	200
Erythromycin	0, 10, 20, 30, 40, 50, 75, 100	150	40	/
Spiramycin	0, 50, 75, 100, 200, 250, 300, 400	-	200	/
Sulfadiazine	0, 25, 50, 75, 100, 150, 200, 300	-	150	<25
Sulfadimidine	0, 50, 75, 100, 200, 250, 300, 400	-	300	50
Lincomycin	0, 25, 50, 75, 100, 150, 200, 300	50	50	/
Danofloxain	0, 50, 75, 100, 200, 250, 300, 400	-	100	/
Enrofloxacin	0, 50, 100, 200, 300, 400, 500, 600	-	400	/


*“-”means no MRL. “/” means not detected. ^*1*^ (The European Commission (2010)). ^*2*^Food, 2015*.

**Table 5:** LODs of microbiological system in honey (3.25 h).

Antimicrobial agents	Spiked levels /(μg/L)	Recommended concentration (RC) (Community Reference Laboratories, 2007) /(μg/L)	This kit /(μg/L)	Premi^®^ Test (Stead et al., 2004) /(μg/L)
Penicillin G	0, 1, 2, 3, 4, 5, 6, 8	-	4	5
Cefquinome	0, 2.5, 5, 10, 20, 40, 60, 80	-	40	25
Neomycin	0, 25, 50, 75, 100, 150, 200, 300	40	50	/
Streptomycin	0, 50, 100, 200, 250, 500, 750, 1000		200	>400
Doxycycline	0, 25, 50, 75, 100, 150, 200, 300	20	100	10
Tetracycline	0, 50, 75, 100, 200, 250, 300, 400		300	10
Erythromycin	0, 10, 20, 30, 40, 50, 75, 100	20	40	15
Spiramycin	0, 50, 75, 100, 200, 250, 300, 400		200	/
Sulfadiazine	0, 25, 50, 75, 100, 150, 200, 300	50	150	25
Sulfadimidine	0, 50, 75, 100, 200, 250, 300, 400		300	25
Lincomycin	0, 10, 20, 30, 50, 75, 100, 150	-	30	25
Danofloxain	0, 50, 75, 100, 200, 250, 300, 400	-	100	/
Enrofloxacin	0, 50, 100, 200, 300, 400, 500, 600	-	200	200


*“-”means no recommended concentration. “/” means not detected*.

### Evaluation Protocol

**FIGURE 1:**
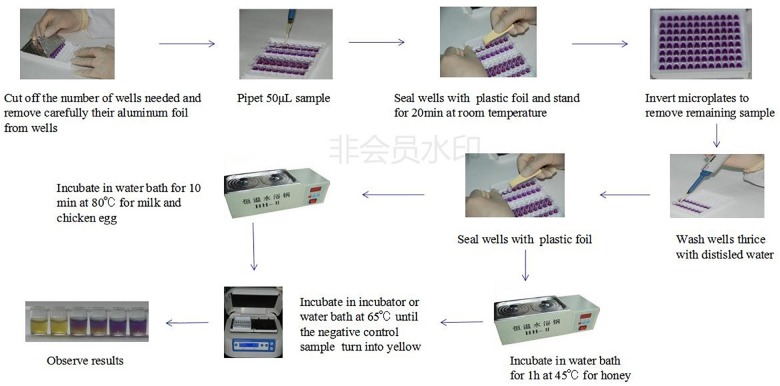
The whole evaluation protocol of the kit.

**FIGURE 2:**
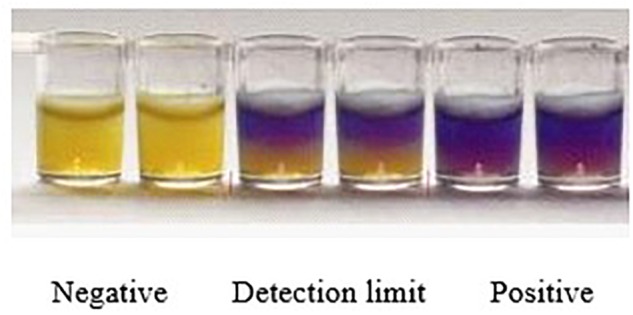
Yellow color indicates negative result, half yellow indicates LOD and purple color shows the positive results.

The whole evaluation protocol of the kit was shown in Figure 1. Firstly, the number of wells in microtiter plates needed were cut off and their aluminum foil were removed carefully from wells. Secondly, a 50 μL control and spiked samples were added into each well of microplates. Thirdly, the microplates were pre-incubated at room temperature (RT) for 20 min to allow the sample to diffuse through the medium. Fourthly, the remaining sample on the microplates medium surface was eliminated by inverting microplates and the wells were washed thrice with distilled water. Fifthly, the wells were sealed with an adhesive sheet and the microplates having milk and chicken egg samples were incubated in water bath for 10 min at 80°C while the microplates having honey samples were incubated in water bath for 1 h at 45°C. Finally, microtiter plates were incubated in microplates incubator at 65°C until the negative control sample had turned into yellow (approximately 3–4 h). The end-point is determined by visually assessing the color change in wells of microtiter plates. During the incubation period, the wells agar bed can be divided into three theoretical vertical zones, a score is assigned to the sample based on the zone color action pattern. An example is presented in Figure 2. 3 zones yellow and 2/3 yellow = negative (-), 1/2 yellow = detection limit (+/-), 2/3 purple and 3 zones purple = positive (+).

### Validation Protocol

#### Limit of Detection (LOD)

The dose–response curves of the antimicrobial agents were established according to the ISO13969: 2003 guidelines. Eight concentrations were prepared with different levels for each drug, and twenty-four replicates were prepared for each concentration. The LOD were estimated as the concentration that was 95% of positive results (International Organizaton for Standardization, 2003).

#### Specificity and Selectivity

One hundred control samples of milk, chicken eggs and honey respectively were analyzed with this kit for the determination of false-positive rate. The sample pre-treatment method was same as described in the “Evaluation Protocol” section. Moreover, the false-positive rate values were calculated as follows:

False-Positive Rate = (Numbers of Positive Samples/Total Control Samples) × 100%

However, one hundred control samples of each animal origin food spiked at the level of interest (MRL or LOD) were analyzed with this kit for the determination of false-negative rate. The method of sample pre-treatment was similar to described in the “Evaluation Protocol” section. Additionally, the false-negative rate values were calculated as follows:

False-Negative Rate = (Numbers of Negative Samples/Total Spiked Samples) × 100%

### Ruggedness

To determine the ruggedness of this kit, the effects of five factors including five different wells in one microplate, five different microplates in same batch, five different batches microplates, two different breeds (buffalo milk, Holstein milk), three different analysts on the false-positive rate, false-negative rate, sensitivity and detection time were evaluated. The ruggedness experiment was repeated three times for each factor. Moreover, the robustness study focused on seven representative antimicrobial agents of seven different kinds of antibiotics. In addition, the ruggedness of the kit was represented by the coefficient of variations (CVs).

### Stability

The kit stability was determined on the basis of appearance, smell, detection capability, detection time, which were evaluated with same batch kits stored at 4°C over 6 months (0, 7, 15, 30, 60, 90, 120, 150, 180 days). The kits stability experiment was performed for three batches kits. Additionally, the validation experiment focused on seven representative antimicrobial agents of different kinds of antibiotics and milk.

### Confirmation by Liquid Chromatography – Tandem Mass Spectrometry (LC/MS-MS)

Seven Holstein cows at the stage of postpartum (between 60 and 90 days) and with a history of no antibiotics exposure in last 9 weeks were raised in an ideal environmental condition of standard temperature and humidity at the dairy farm of HZAU (Wuhan, Hubei, China). The seven cows were treated with PEN, STR, SDZ, LIN, and ENR by intramuscular injection, however, TET and ERY by intravenous injection respectively. Three milk samples from each cow were collected and tested for the presence of antibiotics residues at intervals of 0, 24, 48, 72, and 96 h respectively after drugs administration. All samples were analyzed by the kit in present study as described in the “Evaluation Protocol” section and by a multi-residue LC/MS-MS method (Jank et al., 2017).

## Results

### Detection Capability

The detection capabilities of the kit used in present study against 13 different antibiotics belonging to seven different groups in respective antibiotics diluents was shown in Table 2. It was observed that the LODs of the kit were less than or equal to MRL in milk for β-lactam, aminoglycosides, TET, macrolides, sulfonamides and lincosamides, however, the LODs for DOX and quinolones were higher than MRL in milk.

The LODs of the kit for different kinds of antibiotics in milk were given in Table 3. It was revealed that the LODs of the kit were less than or equal to MRL in milk for β-lactam, aminoglycosides, macrolides, lincosamides. However, the LODs for tetracyclines, sulfonamides and quinolones were higher than MRL in milk.

The detection capability of this kit for different kinds of antibiotics in chicken egg was given in Table 4. There are MRLs only for NEO, TET, ERY, LIN in chicken egg. It indicated that the LODs of this kit for all kinds of antibiotics in chicken eggs were same like determined in milk. Moreover, the LODs for NEO, ERY, LIN were less than or equal to MRL in chicken egg.

The LODs of this kit for various antibiotics in honey were shown in Table 5. In the case of honey, there are no MRLs for antibiotics residues, but the recommended concentration of aminoglycosides, tetracyclines, macrolides, sulfonamides were used as such (Community Reference Laboratories, 2007). It was known that the LODs of this kit for different kinds of antibiotics in honey were similar to those determined in milk. However, the LODs for aminoglycosides, tetracyclines, macrolides, sulfonamides were higher than the recommended concentrations (Community Reference Laboratories, 2007).

### Specificity

Results showed that the false positive rate of this kit used in milk, chicken egg and honey all were 0%. The false-negative rate results of this kit used in milk, chicken egg and honey were given in Tables 6–8. It indicated that the false-negative rate of this kit used in three animal foods all were 0%.

**Table 6:** False negative rates of the kit in milk.

Antibiotics	MRL /(μg/L)	LOD /(μg/L)	Spiked concentration /(μg/L)	Sample numbers	Negative sample numbers	False negative rate/%
Penicillin G	4	2	4	100	0	0
Cefquinome	20	40	40	100	0	0
Neomycin	1500	50	1500	100	0	0
Streptomycin	200	200	200	100	0	0
Doxycycline	0	100	100	100	0	0
Tetracycline	100	300	300	100	0	0
Erythromycin	40	40	40	100	0	0
Spiramycin	200	200	200	100	0	0
Sulfadiazine	100	150	150	100	0	0
Sulfadimidine	100	300	300	100	0	0
Lincomycin	150	120	150	100	0	0
Danofloxain	30	100	100	100	0	0
Enrofloxacin	100	400	400	100	0	0


**Table 7:** False negative rates of the kit in chicken egg.

Antibiotics	MRL /(μg/L)	LOD /(μg/L)	Spiked concentration /(μg/L)	Sample numbers	Negative sample numbers	False negative rate/%
Penicillin G	-	4	4	100	0	0
Cefquinome	-	40	40	100	0	0
Neomycin	500	100	500	100	0	0
Streptomycin	-	200	200	100	0	0
Doxycycline	-	100	100	100	0	0
Tetracycline	200	300	300	100	0	0
Erythromycin	150	40	150	100	0	0
Spiramycin	-	200	200	100	0	0
Sulfadiazine	-	150	150	100	0	0
Sulfadimidine	-	300	300	100	0	0
Lincomycin	50	50	50	100	0	0
Danofloxain	-	100	100	100	0	0
Enrofloxacin	-	400	400	100	0	0


*“-” means no MRL*.

**Table 8:** False negative rates of the kit in honey.

Antibiotics	Recommended concentration (RC) (Community Reference Laboratories, 2007) / (μg/L)	LOD /(μg/L)	Spiked concentration /(μg/L)	Sample numbers	Negative sample numbers	False negative rate/%
Penicillin G	-	4	4	100	0	0
Cefquinome	-	40	40	100	0	0
Neomycin	40	50	50	100	0	0
Streptomycin		200	200	100	0	0
Doxycycline	20	100	100	100	0	0
Tetracycline		300	300	100	0	0
Erythromycin	20	40	40	100	0	0
Spiramycin		200	200	100	0	0
Sulfadiazine	50	150	150	100	0	0
Sulfadimidine		300	300	100	0	0
Lincomycin	-	30	30	100	0	0
Danofloxain	-	100	100	100	0	0
Enrofloxacin	-	200	200	100	0	0


*“-” means no recommended concentration*.

### Ruggedness

Results indicated that three factors of different wells in one microplate, different microplates in same batch, different batches kits had no effect on the ruggedness of the kits. However, different breeds and different analysts had some effect on the ruggedness of kits. Moreover, the CVs of different analysts for false positive rate, false negative rate, detection time, and sensitivity of kits all were less than 4% (see Table 9). In addition, the difference of different breeds among false positive rate, false negative rate, detection time and sensitivity of kits were shown in Table 10. It indicated that the kit in present study showed weaker sensitivity to different kinds of antibiotics in buffalo milk than those determined in Holstein milk with longer detection time. And the false positive and false negative rates of kits used for detecting antibiotics residue in buffalo milk were higher than 0% and less than 5% while the false positive and false negative rates in Holstein milk all were 0%. However, these performances of this kit used in buffalo milk all were up to the standard requirements of residues screening methods.

**Table 9:** The CVs of different analysts for false positive rates, false negative rates, detection time and sensitivity of kits.

Indexes		Different analysts/%
Sensitivity/(μg/L)	Cefquinome	3.4
	Streptomycin	3.4
	Tetracycline	3.0
	Spiramycin	3.7
	Sulfadimidine	3.6
	Lincomycin	3.3
	Enrofloxacin	3.5
False negative rate/%	Cefquinome	3.0
	Streptomycin	3.4
	Tetracycline	3.2
	Spiramycin	3.3
	Sulfadimidine	3.6
	Lincomycin	3.6
	Enrofloxacin	3.5
False positive rate/%		3.8
Incubation time/h		3.6


**Table 10:** False positive and negative rates with detection time and sensitivity of kits in different breeds of milk.

Indexes		Different breeds	
		**Buffalo milk**	**Holstein milk**
Sensitivity/(μg/L)	Cefquinome	45	40
	Streptomycin	220	200
	Tetracycline	320	300
	Spiramycin	220	200
	Sulfadimidine	320	300
	Lincomycin	150	120
	Enrofloxacin	430	400
False negative rate/%	Cefquinome	3	0
	Streptomycin	3	0
	Tetracycline	4	0
	Spiramycin	3	0
	Sulfadimidine	4	0
	Lincomycin	3	0
	Enrofloxacin	4	0
False positive rate/%		4	0
Incubation time/h		3.4	3.0


### Stability

Results showed that the appearance, smell, detection time, detection capability of this kit had no change over 6 months at 4°C. It indicated that the quality guarantee period of the kit is over 6 months.

### Confirmation and Quantification of Incurred Samples by LC/MS-MS

The results of confirmation and quantification of incurred samples by LC/MS-MS was shown in Table 11. It indicated that the samples detected negative with this kit contained antimicrobial drugs residues such as ERY, SDZ, ENR at concentrations lower than LODs of this kit after the LC/MS-MS confirmation. Because LC/MS-MS with a sample pre-treatment of solvent extraction was more sensitive to all kinds of antibiotics than the kit in present study. Additionally, there was no false positive result of the kit. The positive samples, which were confirmed by LC-MS/MS, contained antibiotics residues at concentrations higher than or equal to LODs of this kit. Therefore, the kit in present study was reliable to screen antibiotics residues in incurred samples.

**Table 11:** Results of confirmation of incurred tissues by LC/MS-MS.

Antimicrobial agents	Sample numbers	This kit	LC/MS-MS/ (μg/L)	MRL/ (μg/L)
Penicillin G	15	N(13)	/	4
		P(2)	10	
Streptomycin	15	N(3)	/	200
		P(3)	200	
		P(2)	205	
		P(3)	212	
		P(4)	220	
Tetracycline	15	N(10)	/	100
		P(3)	320	
		P(2)	350	
Erythromycin	15	N(5)	/	40
		N(3)	30	
		P(4)	46	
		P(3)	52	
Sulfadiazine	15	N(2)	/	100
		N(7)	110	
		P(4)	168	
		P(2)	200	
Lincomycin	15	N(10)	/	150
		P(2)	170	
		P(3)	187	
Enrofloxacin	15	N(3)	/	100
		N(5)	200	
		P(4)	400	
		P(3)	450	


*“N” means negative results. “P” means positive results. Numbers in brackets means numbers of negative or positive results. “/” means not detected*.

## Discussion

### Detection Capability

In past years, several microbiological inhibition methods were developed to detect antibiotics in milk. The detection capabilities of different microbiological inhibition methods in terms of test tubes in milk were shown in Table 12. It indicated that the kit in present study was sensitive to β-lactam as previous studies determined. Moreover, the kit was more sensitive to aminoglycosides and macrolides than BRT AIM (Molina et al., 2003), Copan milk test (Le Breton et al., 2007), Delvotest SP-NT (Althaus et al., 2003), Eclipse 100 (Beltrán et al., 2015), Charm Blue Yellow (Linage et al., 2007), and Premi^®^Test (Stead et al., 2004) at MRL levels. Furthermore, several commercial kits such as BRT AIM (Molina et al., 2003), Copan milk test (Le Breton et al., 2007), Delvotest SP-NT (Althaus et al., 2003), Eclipse 100 (Beltrán et al., 2015), Charm Blue Yellow (Linage et al., 2007), and Premi^®^Test (Stead et al., 2004) cannot detect quinolones in milk for that *G. stearothermophilus* is extremely insensitive to quinolones. However, the kit in present study was at least ten times more sensitive to quinolones than previously reported studies (Montero et al., 2005; Linage et al., 2007). And the detection capability of the kit for lincosamides was similar to determined by Delvotest SP-NT (Althaus et al., 2003), Charm Blue Yellow (Linage et al., 2007). Additionally, the LODs for tetracyclines and sulfonamides were slightly higher than Copan milk test (Le Breton et al., 2007), Delvotest SP-NT (Althaus et al., 2003), Charm Blue Yellow (Linage et al., 2007), and Premi^®^Test (Stead et al., 2004).

**Table 12:** The detection capability of different microbiological inhibition methods in term of tubes in milk.

Antibiotics	EU/CODEX MRL in milk /(μg/L)	LOD /(μg/L)						
		The kit	BRT AIM^1^	Copan milk test^2^	Eclipse 100^3^	Delvotest SP-NT^4^	Charm Blue Yellow^5^	Premi^®^ Test^6^
Penicillin G	4	2	2	3	5	2	2	<2.5
Cefquinome	20	40	/	100	/	/	40	/
Neomycin	1500	50	3700	500–2000	9100	100-200	150	/
Streptomycin	200	200	6000	1000	10100	300-500	/	/
Doxycycline	0	100	390	150	260	100	75	100
Tetracycline	100	300	6200	250-500	480	100	100	100
Erythromycin	40	40	630	>200	750	50	150	<100
Spiramycin	200	200	/	>2000	18100	200	500	<125
Sulfadiazine	100	150	5400	50-100	/	50	100	50
Sulfadimidine	100	300	/	100-200	750	25	125	<25
Lincomycin	150	120	/	/	/	100	150	/
Danofloxain	30	100	/	/	/	/	/	/
Enrofloxacin	100	400	/	/	4000	1000-1500	/	/


*^1^Molina et al. (2003); ^*2*^Le Breton et al. (2007); ^*3*^Beltrán et al. (2015); ^*4*^Althaus et al. (2003); ^*5*^Linage et al. (2007); ^*6*^Stead et al. (2004). “/” means not detected*.

Both of chicken egg and honey are important for human health and consumed daily, however, there was few research by microbiological inhibition methods reported about chicken egg and honey. For example, Premi^®^Test is a commercially available kit and widely used for screening of antibiotics residues in milk, muscle, kidney, egg, honey and feed etc. Actually, Premi^®^Test is insensitive to CEF, aminoglycosides, macrolides, LIN and quinolones in chicken egg. However, the kit in present study can detect CEF, aminoglycosides, macrolides, LIN and quinolones in chicken egg, even the LODs for NEO, ERY, LIN were lower than or equal to MRL in chicken egg. Additionally, the LODs of the kit for PEN and DOX were less than or similar to those of Premi^®^Test. But the LODs for tetracyclines and sulfonamides were higher than those determined by Premi^®^Test (Stead et al., 2004). When it comes to honey, the LODs of this kit for β-lactam, ERY, LIN, ENR were less than or similar to determined by Premi^®^Test. Additionally, the kit was more sensitive to aminoglycosides, SPI and DAN than Premi^®^Test. However, Premi^®^Test was more sensitive to tetracyclines and sulfonamides than the kit in present study (Stead et al., 2004).

When compared to previous studies, it was observed that the kit in present study was more sensitive to aminoglycosides, macrolides and quinolones in milk, chicken egg and honey. The CAP can improve the bacteriostatic activity of tetracyclines by synergistic reaction; however, higher concentration of CAP will antagonizes macrolides by competing the subunit 50s site of bacterial ribosomal. Therefore, improvement of the detection capability of the kit in present study for macrolides was operated by lowering CAP concentration in kit’s medium. *G. stearothermophilus var* C953 is only sensitive to β-lactam and lincomycin (Kumar et al., 2012). As a result, in this kit, TMP and CAP was used to improve the sensitivity of the kit to sulfonamides, and tetracyclines separately. At the same time, STR and ENR were used to improve the sensitivity to aminoglycosides, macrolides and quinolones based on the research that improvement of the detection capabilities to ENR by adding moderate concentration of ENR into kits (Shen et al., 2010). A small quantity of STR in the kit can improve the sensitivity of the kit to aminoglycosides and also work with macrolides by synergistic reaction. Even a small amount of STR in this kit can work with tetracyclines by the same reaction principle as tetracyclines do. It was the reason that the kit with high pH value was still sensitive to tetracyclines in antimicrobial agents’s diluents shown in Table 1. Similarly, adding moderate ENR into this kit to improve the detection capability of this kit to quinolones. And the bacteriostatic mechanism of TMP, CAP, STR, and ENR are different, which will produce synergistic reaction, but not antagonism. At same time, the detection capability of this kit to β-lactam and lincosamides was also improved by TMP, CAP, STR, and ENR.

Results showed that the LODs of this kit were less than or equal to MRL in milk for β-lactam, aminoglycosides, tetracyclines, macrolides, sulfonamides, lincosamides, however 1.8–3.4 times MRL in milk for quinolones when the kit in present study was used for screening residual antibiotics in respective antibacterial drugs diluents. However, the LODs of the kit for tetracyclines, sulfonamides and quinolones were higher in milk, chicken egg and honey than determined in respective antibacterial drugs diluents. Moreover, the detection capability of the kit for β-lactam, aminoglycosides, macrolides, lincosamides in milk, chicken egg and honey was same as determined in antimicrobial agents diluents. The reasons can be divided into two aspects: the differences among matrix and the detection capability of the kit in present study. The differences among matrix are pH and matrix components. The matrix’s pH will affect the bacteriostasis effect of all kinds of antibiotics and the detection time of the kit. In addition, the chicken egg, milk and honey are weak alkaline, weak acidic and acidic matrix separately. According to results, the bacteriostasis of all kinds of antibiotics was almost same in chicken egg, milk and honey. Therefore, the pH of matrix was not the main reason. Moreover, the detection time of the kit in the four matrixes were as follows: 3 h for milk; 3.25 h for honey; 3.5 h for chicken egg; 3.75 h for antimicrobial agents diluents. It indicated that the pH of matrix affected the detection time of the kit obviously. The detection time for the matrix with higher pH was longer while the detection time for the matrix with lower pH was shorter. Additionally, compared to antimicrobial agents diluents, the milk, chicken egg and honey are rich in nutrition, which can promote the growth of bacteria in kit’s medium and shorten detection time. It was also reported that dissolution of the final extract in a microbiological growth medium (i.e., Lab Lemco broth) facilitate the bacterial growth cycle and improve the results (Stead et al., 2004). Above all, the main reason maybe that the kit in present study was not enough sensitive to tetracyclines, sulfonamides and quinolones. Because improvement of the detection capability of the kit in present study for macrolides was operated by lowering CAP concentration in kit medium. Moreover, a small quantity of TMP, STR, and ENR in kit medium was adopted to avoid false positive result. Therefore, the bacteriostasis of tetracyclines, sulfonamides and quinolones were weaker with a small quantity of sensitizer such as TMP, CAP, and ENR. Then tetracyclines, sulfonamides and quinolones with sensitizer in kit separately cannot completely inhibit the growth of *G. stearothermophilus* spores in kits. Moreover, the part of the spores produced little acid, which cannot support enough acid for bromcresol purple to change color from purple to yellow under the existing nutritional condition of this kit. Thus, it was shown to be antibioitcs residues positive results of tetracyclines, sulfonamides and quinolones. However, negative results of tetracyclines, sulfonamides and quinolones were indicated when this kit was used for detecting antibiotics residues in milk, chicken egg and honey. Because milk, chicken eggs and honey are rich in nutrition, which made the part of the spores to produce enough acid for bromcresol purple to turn into yellow from purple. Therefore, in the future, further study could be conducted to optimize the kit components such as a mixture of nutrients and sensitizers, and sample pre-treatment methods on the basis of the previous research.

### Specificity

Animal derived food contains natural bacteriostatic substances, which can inhibit the growth of microorganism in microbiological kits and result in false positive results (Gaudin et al., 2013; Billah et al., 2015). In this study, the method of pre-permeation at RT was used to prevent excessive natural bacteriostatic substances in animal food from permeating through the kit’s medium. BRT AIM and Eclipse 100^®^ had used the similar sample pre-treatment method of pre-permeation at 4°C for 1 h (Molina et al., 2003; Montero et al., 2005). But the kit in present study did pre-permeation at RT to shorten the pre-permeation time, and thus shorten the whole operation time of the kit. After pre-permeation, the remaining matrix was poured out and then the microplates were cleaned by water, which will remove the impurities on the microplates medium surface. Finally, a small quantity of natural antimicrobial substances infiltrated into the kit during pre-permeation were denatured by water bath at proper temperature for a certain time, which can avoid the false positive results caused by natural bacteriostatic substances in animal food. The microplates having milk and chicken egg were incubated in water bath for 10 min at 80°C, however, the microplates having honey were incubated in water bath for 1 h at 45°C. High temperature can destroy natural antimicrobial substances in animal food. And the incubation temperature and time for milk and chicken egg were 80°C and 10 min separately. However, enzymes especially amylase in honey are extremely unstable to heating. Therefore, the way of incubation at 80°C for 10 min was not compliant to denature natural antimicrobial substances in honey. And the way of incubation at 45°C for 1 h for honey was decided by optimization experiment. In addition, Schneider and Lehotay (2008) detected antibacterial agents in bovine kidney fluid and serum by Premi^®^Test with similar sample pre-treatment. Microbiological kits were incubated in water bath at 80°C for 10 min after adding samples into test well, which effectively inhibit natural antibacterial substances in animal food. Additionally, microbiological kits heated at proper temperature for little time will not affect the sensitivity of the method (Schneider and Lehotay, 2008).

### Ruggedness

The reproducibility of kits was determined by the experimental materials, preparation process and test operators. Thus, it deserved consideration that the ruggedness of kits in different breeds of animal food, different wells of each microplate, different microplates of same batch, different batches of microplates and different analysts. The CVs of different wells of microplate and different microplates of same batch both were 0%, which indicated that the same standard production process was adopted throughout the whole preparation process of kits. Moreover, the CVs of different batches microplates was also 0%, which revealed that the standard production process was adopted not only throughout the whole preparation process of kits, but also throughout the whole preparation process of *G. stearothermophilus var* C953 spores with the stable performances in kits. The operation results of different operators were not quite different. Because the detection operation flow of this kit was simple with no special training required except the sample procedures according to the instructions the kits. Bovine milk was used as repeatability test because there was a difference in the milk composition of buffalo milk and Holstein milk. Results showed that the false positive rate, false negative rate, detection time and sensitivity were different between buffalo milk and Holstein milk. Because buffalo milk contains more fat, protein and lactose than Holstein milk. Minerals and vitamins in buffalo milk are also dozens of times higher than that of Holstein milk. Therefore, buffalo milk caused more interference to microbiological inhibition methods from matrix than Holstein milk.

### Stability

The stability of kits is important for the transportation, preservation and usage. Results showed that the quality guarantee period of kits was more than 6 months at 4°C. The stability of kits was determined by the production process of kits and the stability of the indicator bacteria. A 150 μL of the culture medium was added into individual wells of microtiter plates using an electronic pipette in a sterile condition. Then these microplates were sealed with aluminized film and stored at 4 °C until use. The purpose of the sealing was to maintain the moisture in kits’ medium and prevent the bacteria and CO_2_ in the environment from contaminating the inner medium. Additionally, *G. stearothermophilus var* C953 spores with stable properties were inoculated into kits during the production process of kits and stored in 4 °C. Moreover, the acid-producing ability of the spore and its sensitivity to antimicrobial agents remained unchanged for a long time. Therefore, the medium of this kit was more stable and the shelf life has been extended.

## Author Contributions

QW, YW, and ZY conceived and designed the experiments. QW, DP, and QL performed the experiments. QW, MS, and AS analyzed the data. QW, ZL, YW, and ZY contributed reagents, materials, and analysis tools. QW wrote the manuscript. All authors discussed the results and commented on the manuscript.

## Conflict of Interest Statement

The authors declare that the research was conducted in the absence of any commercial or financial relationships that could be construed as a potential conflict of interest. The reviewer IM and handling editor declared their shared affiliation.

## References

[B1] Althaus R. Torres A. Peris C. Beltran M. C. Fernandez N. Molina M. P. (2003). Accuracy of BRT and delvotest microbial inhibition tests as affected by composition of ewe’s milk. *J. Food Prot.* 66 473–478. 10.4315/0362-028X-66.3.473 12636303

[B2] Beltrán M. C. Berruga M. I. Molina A. Althaus R. L. Molina M. P. (2015). Performance of current microbial tests for screening antibiotics in sheep and goat milk. *Int. Dairy J.* 41 13–15. 10.1016/j.idairyj.2014.09.007

[B3] Billah M. M. Rana S. M. M. Hossain M. S. Ahamed S. K. Banik S. Hasan M. (2015). Ciprofloxacin residue and their impact on biomolecules in eggs of laying hens following oral administration. *Int. J. Food Contam.* 2:13 10.1186/s40550-015-0019-x

[B4] Community Reference Laboratories (2007). *CRL GUIDANCE PAPER ( 7 December 2007 ). Control Guide Paper.* Berlin: Bundesamt für Verbraucherschutz und Lebensmittelsicherheit.

[B5] Debayle D. Dessalces G. Grenier-Loustalot M. F. (2008). Multi-residue analysis of traces of pesticides and antibiotics in honey by HPLC-MS-MS. *Anal. Bioanal. Chem.* 391 1011–1020. 10.1007/s00216-008-2003-2 18425645

[B6] Food and Agricultures of the United Nations (FAO) and World Health Organization (WHO) (2015). *Maximum Residue Limits (MRLs) and Risk Management Recommendations (RMRs) for Residues of Veterinary Drugs in Foods CAC/MRL 2-2015.* Available at: http://www.codexalimentarius.org/standards/list-of-standards/en/?provide=standards&orderField=fullReference&sort=asc&num1=CAC/MRL

[B7] Gaudin V. De Courville A. Hedou C. Rault A. Diomandé S. E. Creff-Froger C. (2013). Evaluation and validation of two microbiological tests for screening antibiotic residues in honey according to the European guideline for the validation of screening methods. *Food Addit. Contam. Part A Chem. Anal. Control. Expo. Risk Assess.* 30 234–243. 10.1080/19440049.2012.738367 23126529

[B8] International Organizaton for Standardization (2003). *Milk and Milk Products-Guidelines for a Standardized Description of Microbial Inhibition Tests.* Geneva: International Organizaton for Standardization.

[B9] Jank L. Martins M. T. Arsand J. B. Motta T. M. C. Feijó T. C. dos Santos Castilhos T. (2017). Liquid chromatography–tandem mass spectrometry multiclass method for 46 antibiotics residues in milk and meat: development and validation. *Food Anal. Methods* 10 2152–2164. 10.1007/s12161-016-0755-4

[B10] Kumar N. Raghu H. V. Kumar A. Haldar L. Khan A. Rane S. (2012). Spore germination based assay for monitoring antibiotic residues in milk at dairy farm. *World J. Microbiol. Biotechnol.* 28 2559–2566. 10.1007/s11274-012-1065-7 22806162

[B11] Le Breton M. H. Savoy-Perroud M. C. Diserens J. M. (2007). Validation and comparison of the copan milk test and delvotest SP-NT for the detection of antimicrobials in milk. *Anal. Chim. Acta* 586 280–283. 10.1016/j.aca.2006.11.060 17386724

[B12] Linage B. Gonzalo C. Carriedo J. A. Asensio J. A. Blanco M. A. De La Fuente L. F. (2007). Performance of blue-yellow screening test for antimicrobial detection in ovine milk. *J. Dairy Sci.* 90 5374–5379. 10.3168/jds.2007-0245 18024727

[B13] Molina M. P. Althaus R. L. Molina A. Fernández N. (2003). Antimicrobial agent detection in ewes’ milk by the microbial inhibitor test brilliant black reduction test - BRT AiM^®^. *Int. Dairy J.* 13 821–826. 10.1016/S0958-6946(03)00107-9

[B14] Montero A. Althaus R. L. Molina A. Berruga I. Molina M. P. (2005). Detection of antimicrobial agents by a specific microbiological method (Eclipse100^®^) for ewe milk. *Small Rumin. Res.* 57 229–237. 10.1016/j.smallrumres.2004.07.006

[B15] Nagel O. Molina M. P. Althaus R. (2013). Microbiological system in microtitre plates for detection and classification of antibiotic residues in milk. *Int. Dairy J.* 32 150–155. 10.1016/j.idairyj.2013.04.004

[B16] Nagel O. G. Beltrán M. C. Molina M. P. Althaus R. L. (2012). Novel microbiological system for antibiotic detection in ovine milk. *Small Rumin. Res.* 102 26–31. 10.1016/j.smallrumres.2011.11.018

[B17] Schneider M. J. Lehotay S. J. (2008). A comparison of the FAST, Premi^®^ and KISTM tests for screening antibiotic residues in beef kidney juice and serum. *Anal. Bioanal. Chem.* 390 1775–1779. 10.1007/s00216-008-1918-y 18253723

[B18] Shen C. X. Huang X. R. Wu Q. (2010). Assay for the residue of enrofloxacin by sensitized microbial inhibition method. *Fujian Anim. Husb. Vet. Med.* 32 16–18.

[B19] Stead S. Sharman M. Tarbin J. A. Gibson E. Richmond S. Stark J. (2004). Meeting maximum residue limits: an improved screening technique for the rapid detection of antimicrobial residues in animal food products. *Food Addit. Contam.* 21 216–221. 10.1080/02652030310001647280 15195469

[B20] The European Commission (2010). Commission regulation (EU) NO 37/2010 of 22 december 2009 on pharmacologically active substances and their classification regarding maximum residue limits in foodstuffs of animal origin. *Off. J. Eur. Union L* 15 1–72.

